# State and Local Policymakers' Engagement with Neuroscience to Improve Health Policy in the United States

**DOI:** 10.1523/ENEURO.0282-26.2026

**Published:** 2026-10-27

**Authors:** Adam Seth Levine, Debra J. H. Mathews, J. Lomax Boyd

**Affiliations:** ^1^ SNF Agora Institute, Bloomberg School of Public Health, Johns Hopkins University, Baltimore, Maryland 21218; ^2^ Berman Institute of Bioethics, School of Medicine, Johns Hopkins University, Baltimore, Maryland 21205; ^3^ Berman Institute of Bioethics, Johns Hopkins University, Baltimore, Maryland 21205

**Keywords:** health policy, local policymakers, policy engagement, state policymakers

## Abstract

Neuroscience is useful for making progress on public health challenges such as mental health, aging, violence, and addiction. State and local policymakers have substantial authority over these issues but do not always have access to the research and counsel they want. To inform initiatives that could bridge the gap, high-quality information on policymakers' existing and desired engagement is needed. We conducted four nationally representative surveys of local elected policymakers, local public sector staff, state legislators, and state public sector staff to gather this information. Across the four samples, 13.8–28.6% reported recently interacting with a neuroscientist, while 50.3–73.6% reported unmet desire to do so. Prior exposure to disseminated neuroscience information varied, from 19.1 to 68.0% across the samples. Unmet desire for neuroscience-related policy briefs (50.5–76.8%) and news articles (52.7–68.9%) were among the most common for all policymakers. Unmet desire crossed party lines, though with some partisan differences. These findings suggest several avenues to increase evidence-informed health policymaking.

## Significance Statement

Neuroscience is useful for making progress on many public health issues that state and local policymakers have authority over in the United States. Yet policymakers do not always have access to the research they want. We conducted four nationally representative surveys of state and local policymakers (including elected officials and civil servants) and found that there is substantial evidence of unmet desire both to seek counsel from neuroscientists and to receive various forms of disseminated neuroscience information. These findings suggest opportunities to increase evidence-informed policymaking on issues that depend on an understanding of the brain, such as mental health, aging, violence, and addiction.

## Introduction

Some of the most pressing health policy issues we face in the United States—mental health, aging, violence, addiction—share two major characteristics. One is that neuroscience can help inform policy responses (Shonkoff et al., 2009; Altimus, 2017; Greely et al., 2018; Rivara et al., 2019; Perrin et al., 2020; Kenny and Nestler, 2024). The second is that state and local governments have substantial responsibility for designing and implementing policies to address them.

Several examples underscore the value of neuroscience for health policy at the subnational level. For instance, research on early brain development, along with cognitive neuroscientists' engagement with policymakers, directly informed dyslexia prevention policy that is now present in almost all states (Gaab and Duggan, 2024). In addition, research on the negative impact of adverse childhood experiences (ACE's) such as violence and trauma on early brain development informed the creation of California's ACEs Aware Initiative, in which the state reimburses Medi-Cal providers for early screening and response, ensuring that “state and local leaders can align cross-sector efforts for prevention, early detection and effective intervention” (https://osg.ca.gov/wp-content/uploads/sites/266/2020/12/Executive-Summary_Roadmap-for-Resilience_CA_OSG-Report_12092020.pdf). Examples like these are why many researchers are calling for increasing policymakers' access to neuroscience research at all levels of government (Quaglio et al., 2015; Kendall-Taylor and Levitt, 2017; Farah, 2018; Farahany and Ramos, 2020).

Given the importance of neuroscience to several areas of health policy, along with interest in increasing its accessibility to policymakers, it is important to understand the experiences and attitudes of policymakers themselves. We need to know if and how they already access neuroscience research in response to policy challenges they are facing in their communities, as well as if they would like to engage more than they currently are. Moreover, we need to know which dissemination methods they prefer. Gathering this information can directly inform efforts to promote evidence-informed policymaking. To be sure, neuroscience research (as with any scientific research) will rarely (if ever) dictate policy unto itself—economic, ethical, legal, institutional, and political considerations are also important (Jewell and Bero, 2008; Bogenschneider and Corbett, 2021)—yet increasing access to neuroscience research that policymakers want will at least help ensure that our best understanding of the brain informs decision-making.

The present descriptive study analyzes data from four nationally representative surveys of policymakers at the state and local levels. At the state level, this includes one survey of elected state legislators and one survey of state-level civil servants who work on family support services (including social workers, children services specialists, and mental health specialists). At the local level, this includes one survey of elected local legislators and one of local-level civil servants who work on family support services. These policymakers are important to study given the structure of public health responsibility. All states have public health departments, and there over 3,300 local public health departments, with 77% serving one or more counties and the remainder serving one or more municipalities or towns. The large majority (81.4%) are also locally governed (vs units of state government; NACCHO, 2022). Both states and localities are vital locations for health policy innovation (Rutkow et al., 2019).

In each survey, we ask about experiences with and unmet desire for six types of disseminated sources of neuroscience research. We also ask about policymakers' experiences with and desire to engage directly with neuroscientists. We do the latter because past research finds that relationships between trusted researchers and policymakers are important for increasing policymakers' use of research in their work, in addition to the research being relevant, timely, personalized, and accessible (Innvaer et al., 2002; Jewell and Bero, 2008; Oliver et al., 2014; Mullin, 2021; Purtle et al., 2022; Scott et al., 2022).

Overall, our study answers four questions. First, to what extent have policymakers recently engaged with neuroscience research? Second, do policymakers express an unmet desire to engage with neuroscience research more than they currently do? Third, for what issues do they see neuroscience research as most useful in their work? Fourth, focusing on the possibility of directly interacting with neuroscientists, in what ways might they be unsure/hesitant about the value of their counsel?

## Materials and Methods

### Study design

We conducted four nationally representative surveys of policymakers from July 29, 2025, to October 13, 2025. The surveys were fielded by CivicPulse, a nonprofit, nonpartisan organization that maintains large panels of local and state policymakers using publicly available contact information (updated quarterly) from multiple vendors along with information collected through their own research and correspondence.

The total number of policymakers who completed our surveys was 1,346. The sample of local elected policymakers (*N* = 366) is randomly drawn from a comprehensive list of local elected officials in US township, municipality, and county governments serving communities of 1,000 or more. The sample of state legislators (*N* = 267) is randomly drawn from a comprehensive list of legislators in all 50 states. The samples of local and state public sector staff are randomly drawn from national lists of family support service employees associated with local and state governments, respectively. Our sample of local staff includes 363 respondents, and our sample of state staff includes 350 respondents.

CivicPulse recruited all respondents via email (with one initial email and two follow-ups) and used its standard consent language. Compensation was nonmonetary, and all respondents received a research brief from CivicPulse with the findings several months after fielding. Extended Data 1 and Figure 1-3 include question-wording along with details about the construction of survey weights for each sample. This study was approved by the Johns Hopkins Bloomberg School of Public Health IRB and follows the American Association for Public Opinion Research reporting guidelines (AAPOR, 2025).

### Study outcomes

We were mindful that some policymakers may not be very familiar with neuroscience and the overlap of its purview with their work. Given this, before measuring our key outcomes, we included a brief paragraph to make neuroscience understandable to respondents in a way that was accurate and broadly readable. This description read as follows (in which “[decision-makers]” was filled in as appropriate for each sample):

Neuroscience – which refers to the study of how the brain works – may be relevant to a range of policy topics such as addiction, mental health, violence, education, aging, and many others. Some [decision-makers] use findings from neuroscience research in their work, while others may not have thought about neuroscience at all prior to this survey.

Notably, this paragraph also employed language to acknowledge and legitimize that some policymakers may engage with neuroscience research and others may not. From a survey design perspective, our goal was to establish descriptive norms for the full range of experiences and responses to the questions that follow (Tourangeau et al., 2000).

All questions were otherwise the same for each sample. We asked about whether over the past year policymakers engaged with neuroscience research related to their work by interacting directly with a neuroscientist and/or by learning about neuroscience research in one of six possible ways: peer-reviewed articles, news articles, policy briefs, podcasts, lectures/webinars/videos, and blogs. We focused on the past year given that questions about retrospective behavior are more reliable when they specify a well-defined time period that is likely to be meaningful to respondents (Tourangeau et al., 2000). Next, we asked policymakers about their desire to learn about neuroscience research going forward by interacting directly with a neuroscientist and/or via one of the six methods of information dissemination. We report prior engagement in terms of whether respondents had interacted at least once over the past year on at least one decision, and we report unmet desire to engage in terms of whether they want material/interaction “somewhat more than now” or “a lot more than now.” In addition, the surveys asked respondents to indicate in which, if any, of the following policy domains neuroscience research would be useful for their work: addiction, mental health, violence, education, and aging.

One of the core challenges with forming new collaborative relationships between policymakers and neuroscientists is that they often begin as strangers. Policymakers may be uncertain about relationality—whether neuroscientists will relate to them in ways they would like (Levine, 2024). Thus, the surveys asked respondents to identify hesitations they had about interacting with neuroscientists, reflecting possible concerns about the information they might share and what the experience of interacting might be like. Lastly, we measured political ideology, partisanship, gender, age, education, and race/ethnicity. All variables are reported for descriptive purposes; we also present unmet desire results by partisanship.

### Statistical analyses

Summary statistics of respondents' demographic characteristics were calculated. To assess prior engagement, we calculated the average percentage of respondents with any recent engagement with neuroscientists (along with 95% confidence intervals). To assess unmet desire, we calculated the average percentage who wished to engage with neuroscience more than now (with 95% confidence intervals). We did so for all respondents and then by partisanship (and conducted tests of proportions and calculated *z*-scores to examine differences between Democrats and Republicans, and Democrats and Independents). To understand policymakers' hesitations to interact with neuroscientists, we calculated the average percentage of respondents who shared each of eight hesitations (along with 95% confidence intervals). Throughout the paper, all results use survey weights to increase sample representativeness, except where noted (see Extended Data Fig. 1-2 for information on weight construction). Statistical analyses were conducted using Stata 17.

### Limitations

While we account for potential nonresponse by including survey weights for all samples, results may still be biased toward policymakers who are willing to answer a survey on engagement with neuroscience. In addition, and especially when it comes to measuring unmet desire, we acknowledge that answering a survey question is not equivalent to taking action, and respondents who expressed unmet desire in the survey did not have an opportunity to engage in real time. Finally, social desirability is a concern. This is why we included the legitimation language at the beginning of the survey, which is an evidence-based approach to questionnaire design that reduces the impact of social desirability (Tourangeau et al., 2000).

## Results

### Sample characteristics

Our response rates are 11% for local elected policymakers, 8% for local public sector staff, 10% for state legislators, and 10% for state public sector staff. These response rates meet and/or exceed those from other nationwide surveys of state and/or local policymakers recently published in several high impact journals (which are between 7 and 13%; Pagel et al., 2017; Shaffer et al., 2020; Druckman et al., 2023).

Sample characteristics were as follows (all unweighted; Extended Data Fig. 1-1). Among local elected officials, 38.1% identified as Democrats, 32.0% identified as Republicans, 40.4% were female, 86.4% were white, 78.1% had a college degree, and 90.0% are over the age of 45. Among local public sector staff, 44.3% identified as Democrats, 17.2% identified as Republicans, 66.8% were female, 45.0% were white, 80.3% had a college degree, and 61.9% are over the age of 45. Among state legislators, 47.8% identified as Democrats, 45.8% identified as Republicans, 43.0% were female, 85.4% were white, 81.1% had a college degree, and 84.9% are over the age of 45. Among state public sector staff, 39.2% identified as Democrats, 20.2% identified as Republicans, 77.3% were female, 42.2% were white, 84.9% had a college degree, and 54.9% are over the age of 45.

### Recent engagement with neuroscience

Across all four types of policymakers, direct interaction with a neuroscientist was the least common recent type of engagement (with just one exception—slightly more state legislators had directly interacted with a neuroscientist at least once over the past year than had learned about neuroscience research via blogs): 13.8% of local elected officials (95% CI: 10.3, 18.4%), 19.7% of local staff (95% CI: 12.4, 29.8%), 28.6% of state legislators (95% CI: 22.5, 35.6%), and 18.2% of state staff (95% CI: 12.2, 26.2%) reported that they had interacted with a neuroscientist at least once over the past year. In contrast, across all four types of policymakers, learning about neuroscience via news articles was the most common source of engagement: 50.6% of local elected officials (95% CI: 44.8, 56.3%), 60.1% of local staff (95% CI: 50.3, 69.1%), 68.0% of state legislators (95% CI: 60.9, 74.4%), and 52.4% of state staff (95% CI: 43.9, 60.8%) reported that they had done so at least once over the past year (Fig. 1).

**Figure 1.**
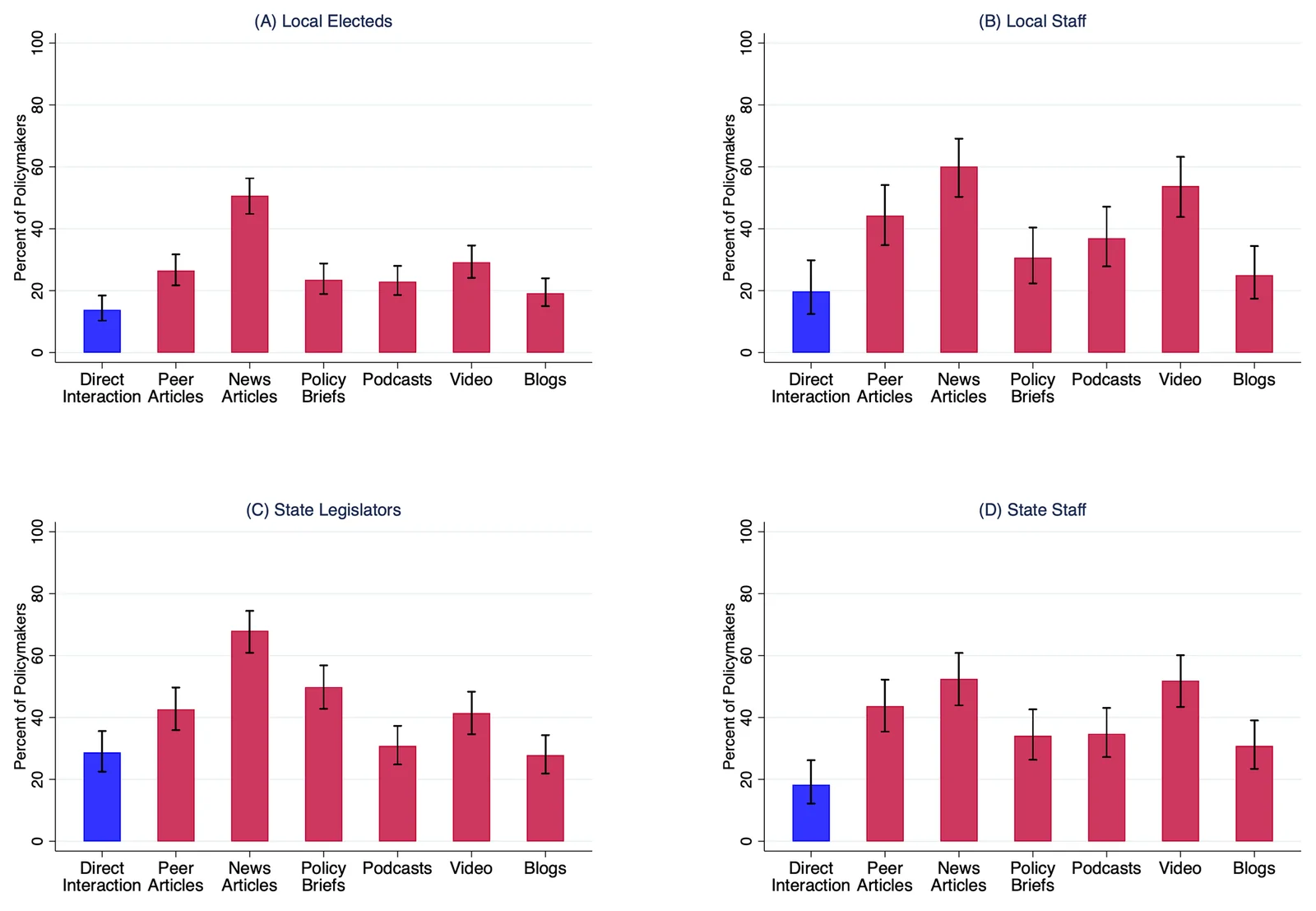
The percentage of state and local policymakers in the United States with prior experience accessing neuroscience research at least once over the past year. Authors' analysis of four nationally representative surveys of local government elected policymakers (*N* = 366), local public sector staff who work in family support services (*N* = 363), state legislators (*N* = 267), and state public sector staff who work in family support services (*N* = 350), along with 95% confidence intervals. Surveys were conducted between July 29, 2025, and October 13, 2025. The figure shows the percentage of respondents who said that they had engaged with neuroscience research at least once over the past year, via direct interaction with a neuroscientist (blue bars) and via six sources of disseminated information (red bars). All data are weighted to increase sample representativeness. Extended Data Figures 1-1 and 1-2 support Figure 1.

10.1523/ENEURO.0282-26.2026.f1-1 Figure 1-1 **Characteristics of respondents in each sample (all unweighted).** Authors’ analysis of four nationally-representative surveys of local government elected policymakers (N=366), local public sector staff who work in family support services (N=363), state legislators (N=267), and state public sector staff who work in family support services (N=350). Surveys were conducted between July 29, 2025 and October 13, 2025. NOTES: Table shows unweighted percentage of respondents with various characteristics. Within each attribute, numbers may not add up to total number of respondents due to respondents answering “other” and/or question non-response. ^a^ These were each separate response categories in the surveys, yet the survey firm grouped them to prevent identifiability. Download Figure 1-1, TIF file.

10.1523/ENEURO.0282-26.2026.f1-2 Figure 1-2 **Further information on sample representativeness.** The randomly-sampled contact lists were balanced on three key Census-based characteristics (proportion of college-educated residents, number of residents, and the presidential vote share from the 2024 election for Trump). This information was supplied by CivicPulse (the survey firm) and used to calculate survey weights. The tables compare the overall sampling frames with the samples collected for these studies. NOTES: All results reported in the main text (unless otherwise noted) use survey weights based on these numbers, provided by CivicPulse and calculated using a post-stratification raking procedure using these three variables. The median value for the local electeds sampling frame is calculated using each individual in the sampling frame, so some counties are repeated. The median sampling frame value for the local public sector staff is calculated using each county in the sampling frame. The median value for the state legislator sampling frame is calculated using each individual in the sampling frame, so some states are repeated. The median sampling frame value for the state public sector staff is calculated using each state in the sampling frame. Download Figure 1-2, DOCX file.

10.1523/ENEURO.0282-26.2026.d1 Data 1 Survey text. Download Data 1, DOCX file.

### Unmet desire for neuroscience

Whereas recent interaction was uncommon, interaction with a neuroscientist was either the most common or second-most common form of engagement on which policymakers expressed unmet desire. 50.3% of local elected officials (95% CI: 44.2, 56.4%), 61.0% of local staff (95% CI: 50.4, 70.8%), 73.6% of state legislators (95% CI: 66.6, 79.6%), and 70.3% of state staff (95% CI: 61.6, 77.7%) expressed a desire to interact with a neuroscientist either “a lot more than now” or “somewhat more than now” to talk about how research findings could apply to policy issues in their work (Fig. 2).

**Figure 2.**
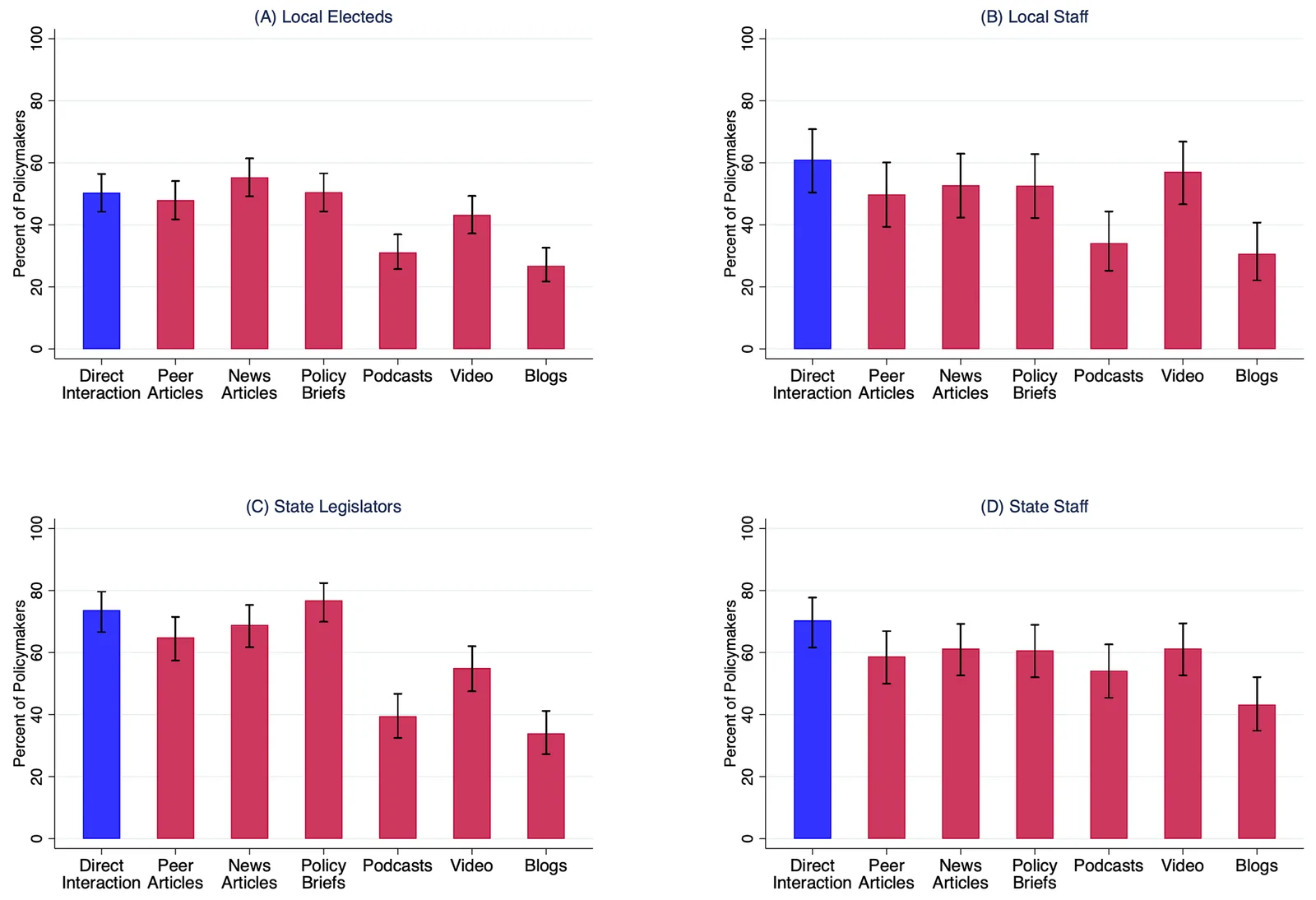
The percentage of state and local policymakers in the United States with unmet desire for neuroscience research. Authors' analysis of four nationally representative surveys of local government elected policymakers (*N* = 366), local public sector staff who work in family support services (*N* = 363), state legislators (*N* = 267), and state public sector staff who work in family support services (*N* = 350), along with 95% confidence intervals. Surveys were conducted between July 29, 2025, and October 13, 2025. The figure shows the percentage of respondents who said that they want to engage with neuroscience research “A lot more than now” or “Somewhat more than now,” via direct engagement with a neuroscientist (blue bars) and to receive one of six sources of disseminated information (red bars). All data are weighted to increase sample representativeness.

Among disseminated sources of information, unmet desire for news articles and policy briefs were two of the highest for all four types of policymakers. 55.3% of local elected officials expressed unmet desire for news articles (95% CI: 49.2, 61.4%) and 50.5% for policy briefs (95% CI: 44.3, 56.6%). Local staff expressed the most unmet desire for video formats (referred to as “lectures/webinars/video” in the survey; 57.0% of local staff; 95% CI: 46.6, 66.8%), and then unmet desire for news articles (52.7%; 95% CI: 42.3, 62.9%) and policy briefs (52.6%; 95% CI: 42.2, 62.8%) was virtually identical. State legislators expressed the most unmet desire for policy briefs (76.8% of state legislators; 95% CI: 69.9, 82.4%) and then second-most for news articles (68.9%; 95% CI: 61.7, 75.3%), and state staff expressed the most desire for news articles, policy briefs, and video (all virtually identical, at ∼61.2, 60.7, and 61.3% of state staff).

### Unmet desire for neuroscience (by party)

Unmet desire for neuroscience crossed party lines (Table 1), with no consistent differences by party that arose across all four types of policymakers. At the state level (both legislators and staff), differences between Democrats and Republicans were observed across almost all forms of engagement, with Democrats expressing higher levels of unmet desire to engage. No differences were observed between Democrats and Independents at the state level.

**Table 1.** The percentage of state and local policymakers in the United States with unmet desire for neuroscience research, by partisanship

Local electeds			
	Democrats	Republicans	Independents
Direct interaction	69.4%	37.8%***	48.8%***
Peer articles	51.7	45.9	50.0
News articles	71.1	51.6***	47.7***
Policy briefs	59.6	46.9	48.4
Podcasts	39.0	31.2	23.5**
Video	49.3	43.3	37.3
Blogs	33.5	26.4	22.2
Local staff			
	Democrats	Republicans	Independents
Direct interaction	73.3%	59.2%	60.5%
Peer articles	62.9	53.0	39.1*
News articles	60.7	56.2	49.3
Policy briefs	67.4	62.0	50.4
Podcasts	48.3	30.3	31.2
Video	70.7	63.9	45.0**
Blogs	40.3	25.7	35.0
State legislators			
	Democrats	Republicans	Independents
Direct interaction	85.1%	62.2%***	78.3%
Peer Articles	70.7	57.3*	57.4
News articles	75.1	62.9*	63.6
Policy briefs	85.8	66.2***	83.5
Podcasts	50.9	27.9***	45.1
Video	60.4	47.3*	78.3
Blogs	46.7	20.0***	30.8
State staff			
	Democrats	Republicans	Independents
Direct interaction	81.8%	64.6%*	73.3%
Peer articles	71.3	43.9**	58.6
News articles	72.5	47.3**	63.2
Policy briefs	73.4	53.7*	61.1
Podcasts	65.4	52.8	54.4
Video	73.4	38.1***	69.6
Blogs	42.8	37.1	51.4

Authors' analysis of four nationally representative surveys of local government elected policymakers (*N* = 366), local public sector staff who work in family support services (*N* = 363), state legislators (*N* = 267), and state public sector staff who work in family support services (*N* = 350), along with 95% confidence intervals. Surveys were conducted between July 29, 2025, and October 13, 2025. Table shows the percentage of respondents who said that they want to engage with neuroscience research “A lot more than now” or “Somewhat more than now,” via direct engagement with a neuroscientist and to receive six sources of disseminated information. Results are presented separately for Democrats, Republicans, and Independents. All data are weighted to increase sample representativeness. Statistical comparisons occur on each row, comparing Republicans to Democrats, and Independents to Democrats; *p* values calculated using two-sided *z* tests. *p < 0.10, **p < 0.05, ***p < 0.01.

At the local level, among local elected officials, those who identify as Democrats are more likely than Independents and Republicans to express an unmet desire to engage directly with a neuroscientist: 69.4% of Democrats expressed unmet desire, as compared with 48.8% of Independents (*z* = −2.58, *p* < 0.01; all statistical comparisons are two-tailed) and 37.8% of Republicans (*z* = −3.94, *p* < 0.01). Similar patterns emerged for local elected officials and their unmet desire for news articles: 71.1% of Democrats expressed unmet desire, as compared with 47.7% of Independents (*z* = −2.91, *p* < 0.01) and 51.6% of Republicans (*z* = −2.50, *p* < 0.01).

### Issues for which neuroscience is useful

The survey asked respondents to indicate which of five issues, if any, neuroscience would be useful for in their work: addiction, mental health, violence, education, and aging. For all types of policymakers, at least one-third reported that neuroscience research would be helpful for their work on all five issues (Fig. 3). The issue on which all types of policymakers were most likely to say that neuroscience research would be useful is mental health, including 60.7% of local elected officials (95% CI: 54.2, 66.8%), 76.5% of local staff (95% CI: 65.7, 84.7%), 83.2% of state legislators (95% CI: 76.8, 88.1%), and 89.3% of state staff (95% CI: 83.8, 93.1%).

**Figure 3.**
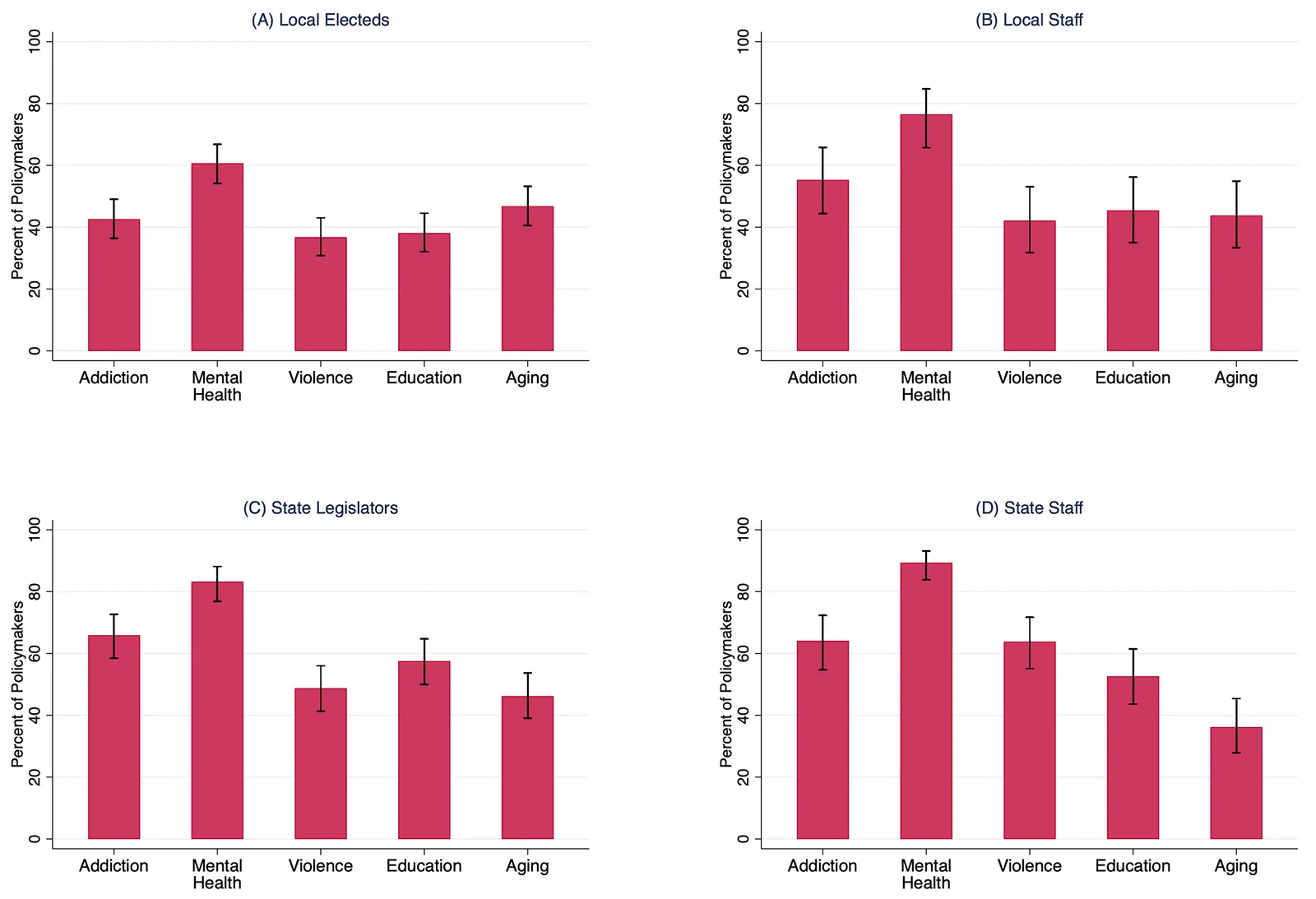
The percentage of state and local policymakers in the United States who view neuroscience research as useful for their work in five health policy domains. Authors' analysis of four nationally representative surveys of local government elected policymakers (*N* = 366), local public sector staff who work in family support services (*N* = 363), state legislators (*N* = 267), and state public sector staff who work in family support services (*N* = 350), along with 95% confidence intervals. Surveys were conducted between July 29, 2025, and October 13, 2025. The figure shows the percentage of respondents who said that neuroscience research would be useful for their work on each of five policy domains. All data are weighted to increase sample representativeness.

### Hesitations about interacting with neuroscientists

With respect to the prospect of engaging anew with a neuroscientist, the surveys asked respondents if they shared any of eight hesitations about interacting with them (Fig. 4). No single hesitation was shared by a majority of policymakers in any sample. Concerns about the information neuroscientists would share (that it may not be practical or that they would use unfamiliar language) were the most common, shared by approximately one-third of policymakers in each sample. Beyond that, approximately one-third of local elected officials and state legislators were concerned that neuroscientists would push a political agenda. Local and state staff were also concerned about neuroscientists being too busy.

**Figure 4.**
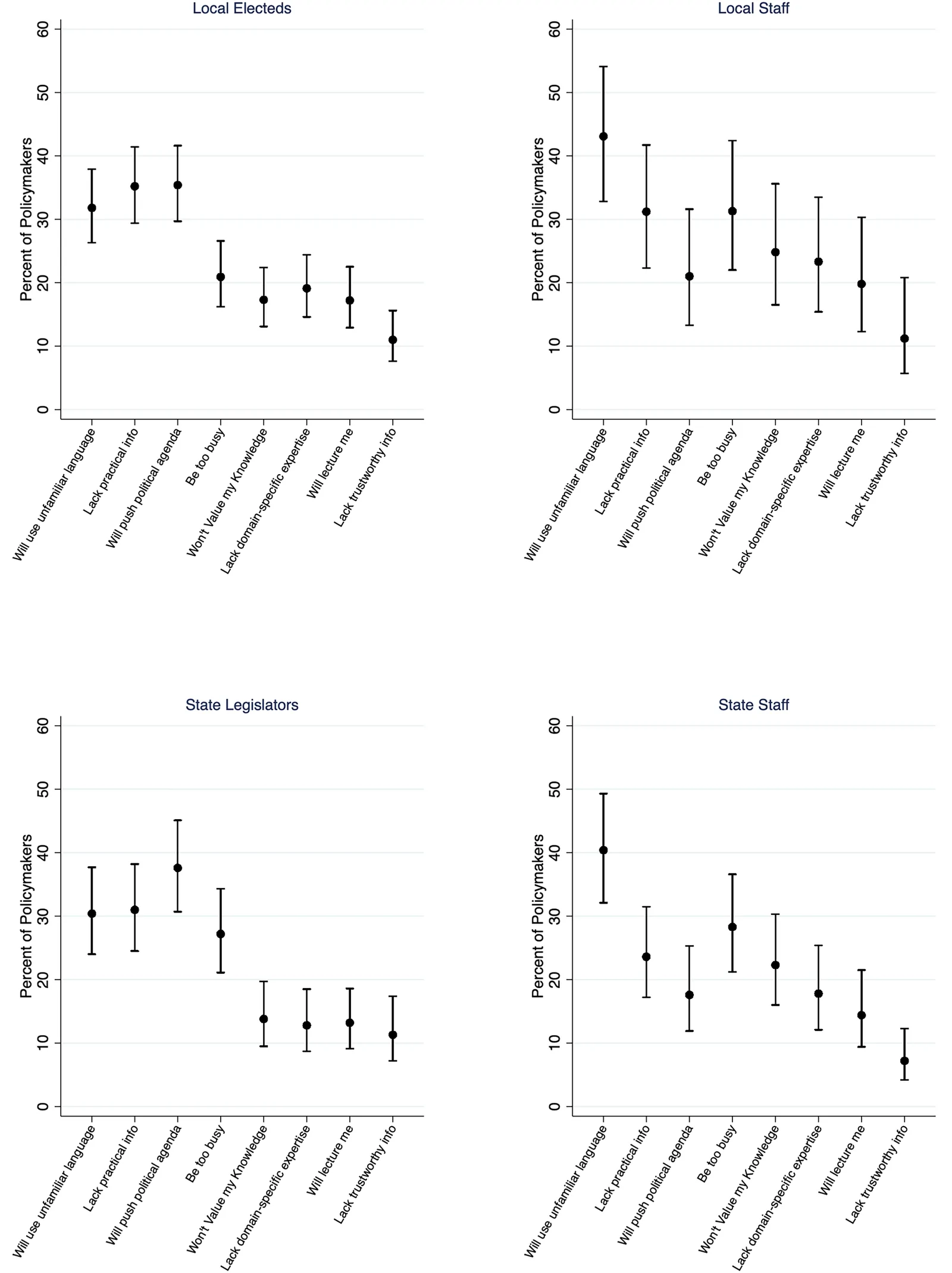
The percentage of policymakers who expressed various concerns about interacting with neuroscientists. Authors' analysis of four nationally representative surveys of local government elected policymakers (*N* = 366), local public sector staff who work in family support services (*N* = 363), state legislators (*N* = 267), and state public sector staff who work in family support services (*N* = 350), along with 95% confidence intervals. Surveys were conducted between July 29, 2025, and October 13, 2025. The figure displays percent who indicated that they shared each hesitation about interacting with a neuroscientist. Error bars indicate 95% CI. All results weighted to increase sample representativeness.

## Discussion

Neuroscience research is important for tackling many public health issues, yet state and local policymakers do not always have access to the research that they would like. Although they rarely seek counsel from neuroscientists in response to policy challenges right now, there is substantial unmet desire to do so. There is also significant unmet desire to learn about policy-relevant neuroscience research via news articles and policy briefs for all four types of policymakers, along with videos for state and local public sector staff.

To be sure, we do not interpret our findings as indicating that majorities of policymakers would pay attention to news articles, policy briefs, and offers to provide counsel at any given moment in time. Competing agenda priorities and limited capacity make that unlikely.

That said, these results suggest a number of key opportunities for those who wish to boost evidence-informed decision-making. Journalists should be encouraged to cover neuroscience advances that are relevant to state and local policy. Those within the research community (e.g., professional associations, academic institutions, individual researchers with neuroscience expertise) should focus on creating policy briefs and other disseminated materials and offering to provide counsel on health policy issues.

In all cases, research findings should be shared in ways that are concise, timely, and relevant to the particular policymakers (Sorian and Baugh, 2002). Communicators should also be mindful of sticking to what the science says and being upfront in situations when the evidence is thin or no consensus exists. For individual researchers, we recognize that some may wish to avoid policy engagement (perhaps because they are too busy, worried about how their research may be used, do not feel confident about engaging, or so on; Grande et al., 2014) and that different incentives, values, time frames, and jargon are important barriers to connecting researchers and policymakers (Brownson et al., 2006). All of this means that when individual researchers are involved, training and support are essential. With that in place, and given that past surveys of scientists find that many *do* express interest in engaging with decision-makers more than they currently are, there is reason to believe that more would engage if given the opportunity (Rose et al., 2020).

Partisan differences are noteworthy, and they echo other work showing that partisanship influences public health policymaking in general (Pacheco et al., 2024). In our case, we should note that policymakers' partisanship is not always visible to outsiders, particularly in unelected roles and also because not all local elections are partisan. Nevertheless, particularly when engaging with elected officials, it is important to be mindful that findings may be viewed through a partisan lens (which also underscores the importance of choosing communicators who can tie research to their audience's values).

Overall, our results suggest that, when working to protect and promote the health of their communities, many policymakers are not currently engaged with neuroscience but would like to be. They would value more counsel from neuroscientists and more opportunities to learn about neuroscience research from disseminated sources, especially via news articles and policy briefs. Those interested in strengthening connections between neuroscience and health policy should be encouraged to and supported in engaging with policymakers to ensure that decisions are informed by the best available evidence of how the brain works.

## Synthesis

Reviewing Editor: Mihaela Iordanova, Concordia University - Loyola Campus Decisions are customarily a result of the Reviewing Editor and the peer reviewers coming together and discussing their recommendations until a consensus is reached. When revisions are invited, a fact-based synthesis statement explaining their decision and outlining what is needed to prepare a revision will be listed below. The following reviewer(s) agreed to reveal their identity: NONE.

This paper presents results from 4 nationally-representative surveys of policy makers about their engagement with neuroscience research. The surveys were carefully constructed, samples were nationally representative, analysis is straightforward and thoughtful, the results are clearly presented and the manuscript is well organized, well written, well-sourced, and careful.

This is one of the rare occasions where the reviewer has little to do but to commend the authors. Although this is not a typical topic for our journal, I have no doubt this data will be of interest to SFN members, SFN staff, and to the various committees within SFN who work on engagement and policy. Therefore, I think eNeuro is an ideal venue, where this paper is most likely to be noticed and utilized by SFN membership.

I appreciate the care that went into the survey instrument. The authors report weighted results, as are appropriate, and all statistical analyses are straightforward and sound. The authors acknowledge limitations (e.g. the fact that reporting a desire for more engagement is not as strong of an indicator of unmet need as actual behavior/action). The discussion is thoughtful, and provides some useful suggestions and sources for SFN members who might want to become more engaged in policy. It's a shame response rates were low, but the authors are correct in pointing out that this is standard for survey research (and much higher than what phone-based surveys and other approaches currently reach). In my opinion, this is an excellent project and an outstanding manuscript; it can be published as is.

I have only one very minor comment: Line 181 - "evidence-based approach" - rather begs for a citation
