# Supplementary material for: State and Local Policymakers' Engagement with Neuroscience to Improve Health Policy in the United States
Source: eNeuro. 2026 Oct 27;13(10):ENEURO.0282-26.2026. doi: 10.1523/ENEURO.0282-26.2026 (PMC13719253; doi:10.1523/ENEURO.0282-26.2026)
Supplement: Supplementary file 1 [file eneuro-13-ENEURO.0282-26.2026-s002.docx]

**Figure 1-2**

Characteristics of respondents in each sample (all unweighted).

*Note: Within each attribute, numbers may not add up to total number of respondents due to respondents answering “other” and/or question non-response.*

| **Attribute** | **Local electeds, No. (%)** | **Local staff, No. (%)** | **State legislators, No. (%)** | **State staff,**  **No. (%)** |
| --- | --- | --- | --- | --- |
| Sex |  |  |  |  |
| Female | 101 (40.4) | 167 (66.8) | 86 (43.0) | 204 (77.3) |
| Male | 149 (59.6) | 83 (33.2) | 114 (57.0) | 60 (22.7) |
| Partisanship |  |  |  |  |
| Democrat | 94 (38.1) | 108 (44.3) | 96 (47.8) | 101 (39.2) |
| Independent | 74 (30.0) | 94 (38.5) | 13 (6.5) | 105 (40.7) |
| Republican | 79 (32.0) | 42 (17.2) | 92 (45.8) | 52 (20.2) |
| Race and ethnicity |  |  |  |  |
| Black, Asian, Hispanic, Native American, Middle Eastern, or Mixed Race^a^ | 34 (13.7) | 137 (55.0) | 29 (14.7) | 152 (57.8) |
| White | 215 (86.4) | 112 (45.0) | 169 (85.4) | 111 (42.2) |
| Education |  |  |  |  |
| College degree | 196 (78.1) | 200 (80.3) | 163 (81.1) | 225 (84.9) |
| No college degree | 55 (21.9) | 49 (19.7) | 38 (18.9) | 40 (15.1) |
| Age |  |  |  |  |
| Born 1920-1965 | 140 (61.1) | 42 (18.2) | 99 (51.6) | 36 (14.8) |
| Born 1966-1980 | 66 (28.8) | 101 (43.7) | 64 (33.3) | 98 (40.2) |
| Born 1981-present | 23 (10.0) | 88 (38.1) | 29 (15.1) | 110 (45.1) |
